# HIV among men who have sex with men in Malawi: elucidating HIV prevalence and correlates of infection to inform HIV prevention

**DOI:** 10.7448/IAS.16.4.18742

**Published:** 2013-12-02

**Authors:** Andrea L Wirtz, Vincent Jumbe, Gift Trapence, Dunker Kamba, Eric Umar, Sosthenes Ketende, Mark Berry, Susanne Strömdahl, Chris Beyrer, Stefan D Baral

**Affiliations:** 1 Center for Public Health and Human Rights, Department of Epidemiology, Johns Hopkins Bloomberg School of Public Health, Baltimore, MD, USA; 2 Department of Emergency Medicine, Johns Hopkins Medical Institute, Baltimore, MD, USA; 3 Department of Community Health, University of Malawi, College of Medicine, Blantyre, Malawi; 4 Centre for Global Health, Trinity College, Dublin, Ireland; 5 Centre for the Development of People, Blantyre, Malawi

**Keywords:** HIV, men who have sex with men (MSM), behavioural risks, stigma, Malawi, prevention

## Abstract

**Introduction:**

There are limited data characterizing the burden of HIV among men who have sex with men (MSM) in Malawi. Epidemiologic research and access to HIV prevention, treatment and care services have been traditionally limited in Malawi by criminalization and stigmatization of same-sex practices. To inform the development of a comprehensive HIV prevention intervention for Malawian MSM, we conducted a community-led assessment of HIV prevalence and correlates of infection.

**Methods:**

From April 2011 to March 2012, 338 MSM were enrolled in a cross-sectional study in Blantyre, Malawi. Participants were recruited by respondent-driven sampling methods (RDS), reaching 19 waves. Trained staff administered the socio-behavioural survey and HIV and syphilis voluntary counselling and testing.

**Results:**

Crude HIV and syphilis prevalence estimates were 15.4% (RDS-weighted 12.5%, 95% confidence interval (CI): 7.3–17.8) and 5.3% (RDS-weighted 4.4%, 95% CI: 3.1–7.6), respectively. Ninety per cent (90.4%, unweighted) of HIV infections were reported as being previously undiagnosed. Participants were predominantly gay-identified (60.8%) or bisexually identified (36.3%); 50.7% reported recent concurrent relationships. Approximately half reported consistent condom use (always or almost always) with casual male partners, and proportions were relatively uniform across partner types and genders. The prevalence of perceived and experienced stigma exceeded 20% for almost all variables, 11.4% ever experienced physical violence and 7% were ever raped. Current age >25 years (RDS-weighted adjusted odds ratio (AOR) 3.9, 95% CI: 1.2–12.7), single marital status (RDS-weighted AOR: 0.3; 95% CI: 0.1–0.8) and age of first sex with a man <16 years (RDS-weighted AOR: 4.3, 95% CI: 1.2–15.0) were independently associated with HIV infection.

**Conclusions:**

Results demonstrate that MSM represent an underserved, at-risk population for HIV services in Malawi and merit comprehensive HIV prevention services. Results provide a number of priorities for research and prevention programmes for MSM, including providing access to and encouraging regular confidential HIV testing and counselling, and risk reduction counselling related to anal intercourse. Other targets include the provision of condoms and compatible lubricants, HIV prevention information, and HIV and sexually transmitted infection treatment and adherence support. Addressing multiple levels of HIV risk, including structural factors, may help to ensure that programmes have sufficient coverage to impact this HIV epidemic among MSM.

## Introduction

Recent years have witnessed an increased awareness of the high burden of HIV among men who have sex with men (MSM) across the globe [1–3]. Emerging research suggests a greater transmission efficiency of HIV through receptive anal intercourse that is approximately 18 times higher than that of penile-vaginal sexual contact, increasing the risk among MSM for acquisition of HIV during sexual intercourse [4,5]. National HIV strategies and funding priorities, however, remain inequitable in many countries [6,7], particularly where structural factors, such as the criminalization of homosexuality, play critical roles in the level of research and programming available to MSM [8,9].

The HIV response in Malawi has focused on the prevention of heterosexual and vertical transmission of HIV to counteract the observed HIV incidence rates of 2–4% among adults in the 1990s. Today, the epidemic remains a generalized one, with an estimated 8.0% HIV prevalence among adult men [10]. Like neighbouring countries, assessments of specific risk factors for the acquisition and transmission of HIV, including transmission among MSM and other populations such as female sex workers, have been limited in the country [11]. Criminalization and stigmatization of homosexuality, as in other settings [8,12,13], are likely underlying factors for the limited targeted research and programming in the Malawian context.

To our knowledge, only two studies in Malawi have assessed sexual and social exposures that place MSM at risk for HIV infection. In 2008, our research team conducted a rapid HIV screening and socio-behavioural assessment among 201 MSM in Lilongwe and Blantyre, Malawi, as part of a comprehensive study across Southern African countries, including Namibia and Botswana, where homosexuality is criminalized [14]. This study documented HIV prevalence at approximately 21% [14], individual risk for HIV infections associated with increased age of the participant and inconsistent condom use [14] and high levels of violence and perceived stigma [15].

Another exploratory study examined socio-demographic and sexual behaviour characteristics among 97 MSM in central and southern Malawi. Although HIV prevalence was not assessed, the study found evidence of high-risk behaviours such as inconsistent condom use (32.5%), transactional sex (23.7%), low exposure to HIV messaging (17.5%) and a low history of HIV testing (58.8% ever tested) [16]. Although these studies were the first and only to elucidate the socio-behavioural factors among MSM in Malawi, they were rapid assessments and served to highlight areas for future research and prevention.

In response to the global epidemic of HIV among MSM, combination prevention packages have been put forth as a key method to curb the HIV epidemic among MSM [17,18]. To inform the content and scale of a combination HIV prevention intervention (CHPI) for MSM in Malawi, we conducted this study to estimate HIV prevalence, characterize associations of prevalent HIV infections, and evaluate barriers and facilitators to uptake of HIV prevention services among MSM in Blantyre, Malawi. Research was conducted in collaboration with a community-based organization, the Centre for the Development of People (CEDEP), and the Malawi College of Medicine, University of Malawi.

## Methods

### Study population and setting

This cross-sectional assessment was conducted from August 2011 to March 2012 in Blantyre, Malawi. Eligibility requirements for participation included being born male, being aged 18 years or older, being fluent in Chichewa or English, having reported anal sex with another man in the last 12 months, having no prior participation in this study, and providing informed verbal consent to participate. Study activities were conducted in private rooms of CEDEP's study site and implemented by staff from CEDEP, which provides HIV prevention activities for MSM in Malawi, and the Malawi College of Medicine. All staff members were trained in confidentiality and human subjects protection, qualitative and survey research and respondent-driven sampling (RDS) methods.

### Recruitment method

Participants were recruited via RDS, a chain recruitment method often used to achieve more representative samples of hard-to-reach populations [19]. Recruitment began with 10 purposively selected seeds who were each provided with three study-specific coupons with which to recruit peer MSM from their social network into the study. Initiation of seeds was staggered over the duration of the study, taking into consideration potential propagation failure by some seeds and eventual die-out of the chains. Seeds were recruited from the pool of MSM who were involved in local HIV prevention programmes or had participated in prior formative research, and they were selected to represent a range of characteristics, including age, education, employment and sexual identity. Individuals who were recruited by seeds and enrolled in the study were then provided with three study coupons for further recruitment of peers. This process continued until the target sample size was reached. Participants were reimbursed K1500.00 (US$5.00) for transportation costs for participation in the study and K500.00 (US$1.50) for recruitment of each peer into the study. A full description of traditional RDS methodology can be found elsewhere [20]. Netdraw software (Analytic Technologies) was used to monitor RDS recruitment [21].

### Sample size

The sample size calculation was powered on the assumed 85% effectiveness of condoms in preventing the transmission of HIV during intercourse [22]. Thus, we assumed that approximately 30% of the sample would be consistent condom users and that they would be 85% less likely to be living with HIV than the 70% who are not consistent condom users. Based on previous research, we estimated that the HIV prevalence in the population would be about 20%, equating to 27% among non-consistent condom users, 4% among consistent condom users and a 30% population prevalence of consistent condom usage. We used a design effect of 1.5 [23], power set at 80% and a significance level of 95%, which resulted in an effective sample size estimate of 345 participants for which we had targeted 350 MSM.

### Measures

Participation included a structured survey instrument and a biological assessment of HIV and syphilis. Trained interviewers administered surveys in the Chichewa language, following pilot testing. Measures included sociodemographic characteristics, substance use, mental health and depression symptoms, sexual relationships and disclosure of orientation or sexual practices to family and peers. Measures of sexual practices included practices with men and women, including anal, oral and vaginal sex; number of sexual partners and partner characteristics; concurrent relationships, defined as “two sexual partnerships at the same time or two ongoing sexual partnerships (male and/or female genders)”; and transactional sex (purchased or sold). We measured HIV knowledge and prevention, including aspects of condom and condom-compatible lubricant use; HIV testing and counselling exposures; and access to and uptake of health services. Human rights measures included experiences of physical and sexual violence, experienced and perceived stigma and history of imprisonment. Recall periods were lifetime, last 12 months or both, and they are specified in the results tables. The development of survey questions, recruitment methods, coupons and study procedures was informed by formative research that was conducted in May–July, 2011 [24].

### Biologic specimens

Following completion of the interview, participants proceeded to HIV and syphilis testing. A trained nurse from the College of Medicine conducted HIV testing, blood specimen collection and pre- and post-test counselling. Blood-based rapid HIV tests were conducted simultaneously using the Determine^®^ HIV-1/2 and Uni-Gold rapid HIV tests (manufactured, respectively, by Inverness Medical, Chiba, Japan; and Trinity Biotech, Bray, Ireland). Participants received their HIV test results and post-test counselling within 15 minutes of collection. Separate specimens were collected for confirmatory testing of discrepant or indeterminate HIV rapid tests using Western blot in accordance with Malawian National Guidelines [25]. Approximately 5 ml of whole blood was collected for TPHA (treponema pallidum haemagglutination test) syphilis testing (Bio-rad, Hercules, CA, USA). Resource constraints prevented the use of the nontreponemal test, which would differentiate active from past syphilis infections. Confirmatory HIV and syphilis tests were analysed at the Malawi College of Medicine laboratory in Blantyre. Participants returned within one to two weeks to receive their syphilis test results. Participants testing positive for HIV and/or syphilis were referred to the local hospital or to the Johns Hopkins antiretroviral therapy and sexually transmitted infection clinic located at Queens Hospital. Participants were provided with information about local health centres that had, as part of the study, received training for the provision of services to MSM. One trained team member (EU) provided counselling services to MSM participants as needed.

### Analysis

Johns Hopkins University conducted secondary data analysis of collected data. The principal outcome of interest was HIV diagnosis with predictor variables that included demographics (education, age, number of children and marital status), socio-economic variables, lifetime residence in urban or rural locations, recent sexual behaviours, human rights exposures, HIV prevention methods, health-seeking behaviour and laboratory markers of syphilis infection. Variable-specific individualized weights, which take into account estimates for individual degrees, were computed by a data-smoothing algorithm using RDS for Stata [26]. The estimated weights were used in univariate RDS-weighted analyses. HIV status individualized weights were used in the bivariate and multivariate RDS-weighted analyses. Bootstrapped 95% confidence intervals (CIs) were computed using 1000 iterations for the estimated descriptive statistics [23]. Homophily, a measure of to what extent respondents prefer to recruit from their own group rather than at random, was estimated where appropriate and presented in the results in Table 1.

**Table 1:** Demographics, identity and health characteristics of RDS-recruited MSM in Blantyre, Malawi (August 2011–March 2012; *n=*338)

		Crude		RDS weighted		
			
Variable	Categories	*n*	(%)	(%)	(95% CI)	Homophily
**Demographics**						
Age	18–25 years	192	56.8	58.5	[50.2–66.8]	0.261
	≥26 years	146	43.2	41.5	[33.2–49.8]	0.231
Highest level of education (completed)	Less than secondary	146	43.2	46.3	[38.9–53.8]	0.115
	Secondary or higher	192	56.8	53.7	[46.2–61.1]	0.147
Employment status	Unemployed	158	46.8	51.7	[44.2–59.3]	0.008
	Employed	136	40.2	36.8	[29.6–44.1]	0.153
	Student	44	13.0	11.5	[0.74–15.5]	0.044
Gender identity	Male	263	77.8	80.2	[74.8–85.6]	0.008
	Female	65	19.2	17.0	[11.8–22.1]	0.153
	Transgender	10	2.9	2.8	[0.8–4.9]	0.044
Sexual orientation	Gay or homosexual	210	62.3	60.8	[53.6–68.1]	0.092
	Bisexual	125	37.1	36.3	[29.1–43.4]	0.070
	Heterosexual or straight	2	0.6	2.9	[0.0–6.7]	−0.029
Marital status (with a woman)	Married or cohabiting	54	16.0	16.2	[9.7–22.7]	0.196
	Single, divorced or separated	284	84.0	83.7	[77.4–90.1]	0.255
Number of children	None	285	84.6	84.9	[78.2–91.6]	0.224
	One or more	52	15.4	15.1	[0.8–21.8]	0.142
Type of location where majority of life was spent	Urban	279	82.5	78.3	[72.1–84.6]	0.231
	Rural	59	17.5	21.7	[15.4–27.9]	0.050
**Health indicators**						
HIV diagnosis	Negative	286	84.6	87.5	[82.2–92.8]	−0.090
	Positive	52	15.4	12.5	[7.2–17.8]	0.022
Syphilis diagnosis	Negative	319	94.7	95.6	[92.9–98.3]	−0.195
	Positive	18	5.3	4.4	[1.7–7.1]	0.009

To develop the statistical model, we first carried out bivariate analysis to assess the association of HIV status with the control variables (Table 4). Demographic variables were included in the multivariate logistic regression model regardless of the estimated strength of their bivariate association with HIV status. Selected non-demographic variables were included in the multivariate model if the chi-square *p*-value of association with HIV status was ≤0.25. Some variables such as HIV testing were not included in the multivariate model due to collinearity. The final model, presented in Table 4, includes demographics and variables left in the final model following goodness-of-fit tests. All statistical analyses were conducted using Stata 12.1 [27]. Results provided in the text report RDS-weighted estimates (unless otherwise specified), while tables display unweighted and RDS-weighted estimates as well as 95% CIs for weighted estimates. Table 4 presents the results of bivariate and final multivariate analyses, including unweighted and RDS-weighted odds ratios (ORs) and adjusted ORs (AORs) for the final multivariate model.

### Human subjects

Research activities were reviewed and approved by the Malawi College of Medicine Ethics and Research Committee and for secondary analysis by the Johns Hopkins Bloomberg School of Public Health Institutional Review Board.

## Results

**Figure 1:**
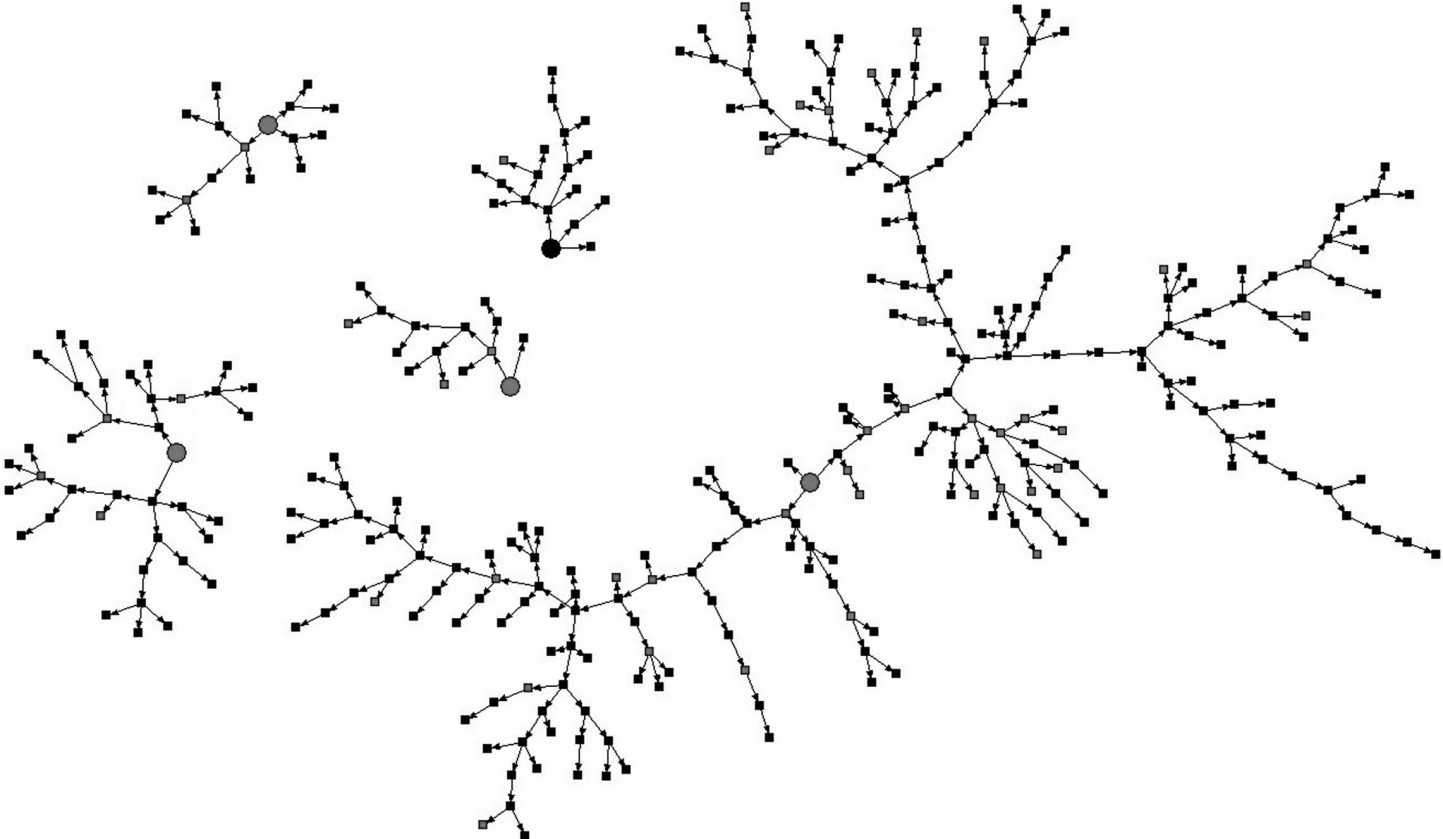
RDS recruitment diagram from 338 MSM recruited in Blantyre, Malawi. Large nodes=seeds; small nodes=recruits; grey=HIV+ according to Determine rapid HIV test; black=HIV- on Determine rapid HIV test.

A total of 338 MSM (including original seeds) were recruited via RDS and enrolled into the study, reaching 19 waves of recruitment. Out of 10 seeds, five recruited participants; one recruitment chain was responsible for the recruitment of 70% of the study population. Three recruitment chains are reflective of later seed initiation. A total of 706 coupons were distributed with a return rate of 48%. The majority of participants reported recruitment by a friend (60.5%) or sex partner (32.3%). Median MSM network size was 8 (range 1 to 800). Figure 1 displays the RDS recruitment diagram, highlighted by HIV diagnosis. We used this method to monitor recruitment and to assess whether HIV diagnosis inhibited recruitment, which appeared not to be the case.

Participants’ median age was 25.1 years (range: 18 to 49). Based on RDS-weighted estimates, 51% were unemployed and 21.6% had ever been in jail or prison. Eighty per cent identified as male gender. Sixty-one per cent identified as gay or homosexual, and 36.3% reported bisexual identity. Sixteen per cent were married or cohabitating with a woman. Table 1 displays sociodemographic characteristics.

The crude prevalence of HIV infection in this population was 15.4%, with an RDS-weighted estimate of 12.5% (Table 1). The majority, 90.4% (unweighted), of these infections were previously undiagnosed; these participants had either self-reported as negative or reported never being tested for HIV infection. Positive syphilis diagnosis was low at 4.4%.

Table 2 presents sexual practices, partner characteristics and social exposures. Only 18.1% had ever disclosed sexual practices or orientation to their family, and equally few (18.9%), had ever disclosed to a health provider. Participants reported a mean of four male partners (range: 1 to 50), and 31% reported having female partners in the last 12 months. Half of the population reported concurrent sexual relationships, and, among those in a relationship, 61.3% believed their partner was also involved in a concurrent relationship. Prevalence of perceived and experienced stigma and discrimination exceeded 20% of the population for almost all variables, 7.0% were ever raped and 11.4% had ever experienced physical violence.

**Table 2:** Sexuality, partnerships and risk exposures of RDS-recruited MSM in Blantyre, Malawi (August 2011–March 2012; *n=*338)

		Unweighted		RDS weighted	
			
Variable	Categories	*n*	(%)	(%)	(95% CI)
Ever disclosed sexual practice to family	Yes	69	20.4	18.1	[12.9–23.2]
Ever disclosed sexual practice to health provider	Yes	70	20.8	18.9	[13.3–24.5]
Gender of first sex partner	Male	226	67.1	69.4	[62.5–76.3]
	Female	111	32.9	30.6	[23.7–37.5]
Age at first sex with another man	<16 Years old	55	16.3	14.9	[8.9–21.0]
	16–20 Years old	174	51.6	53.1	[45.5–60.8]
	21–25 Years old	74	22.0	22.4	[16.1–28.8]
	>25	34	10.1	9.6	[5.3–13.9]
Sexual partners in last 12 months: mean (range)	Male partners [*n=*334]	3.8	(1–50)	N/A	N/A
	Female partners [*n=*107]	2.9	(1–20)	N/A	N/A
Concurrent relationships, last 12 months*	None	160	47.3	49.5	[42.3–56.8]
	Yes, two or more male and/or female partners	178	52.7	50.7	[43.3–57.9]
Believes partner has concurrent relationship* (*n=*76)	Yes	59	77.6	61.3	[30.1–90.9]
Normally has sex with men in a private home	Yes	224	66.3	67.6	[60.5–74.8]
… in bar or clubs	Yes	98	29.0	26.1	[20.2–32.1]
… in a hotel	Yes	78	23.1	20.1	[14.2–26.0]
**Social exposures**					
Ever felt excluded from family gatherings	Yes	87	25.7	26.1	[20.0–32.2]
Ever felt rejected by friends	Yes	118	35.0	27.2	[20.7–34.]
Feels there are safe places to go to socialize with other MSM	Yes	223	66.8	66.0	[59.1–72.9]
Ever felt afraid to seek health services	Yes	68	20.1	21.5	[15.1–27.9]
Ever been in jail or prison	Yes	74	22.1	21.6	[14.1–29.1]
Ever experienced physical violence	Yes	40	11.8	11.4	[6.1–16.8]
Ever raped (*N*=337)	Yes	26	7.7	7.0	[3.5–10.5]

* Concurrent sexual partnership: two or more male or female sexual partners during the same time period.

Responses to questions on knowledge of HIV risk, prevention methods and practices are reported in Table 3. Approximately half of the participants with casual male partners (*n*=256) reported using condoms always or almost always with casual male partners; frequencies were approximately similar across partner type (e.g., casual or main) and partner gender. Approximately 44.3% had never been tested for HIV. Among those ever tested for HIV infection, 45.5% (unweighted) had not been tested within the last year.

**Table 3:** Knowledge of HIV risk, prevention methods and practices of RDS-recruited MSM in Blantyre, Malawi (August 2011–March 2012; *n=*338)

		Unweighted		RDS weighted	
			
Variable	Categories	*n*	(%)	(%)	(95% CI)
Condom use in last sex with *main* male partner (*n=*316)	Yes	119	63.0	59.6	[51.7–67.4]
Condom use in last sex with *casual* male partner (*n=*256)	Yes	174	68.0	66.3	[57.4–75.2]
Condom frequency with *main* male partners (*n=*316)	Never	44	14.1	15.3	[9.9–20.6]
	Almost never	52	16.7	18.4	[11.7–25.0]
	Sometimes	63	20.2	19.9	[13.8–25.9]
	Almost always	41	13.1	10.8	[6.3–15.2]
	Always	112	35.9	35.7	[27.9–43.6]
Condom frequency with *casual* male partners (*n*=256)	Never	34	13.3	15.6	[9.5–21.7]
	Almost never	30	11.7	14.0	[6.2–21.9]
	Sometimes	53	20.7	21.4	[14.1–28.6]
	Almost always	28	10.9	4.8	[1.8–7.8]
	Always	111	43.4	44.2	[34.5–53.8]
Condom use at last sex with *main* female partner (*n=*101)	Yes	45	44.5	50.3	[23.9–76.6]
Condom use at last sex with *casual* female partner (*n=*71)	Yes	46	64.8	58.2	[27.5–88.8]
Condom frequency with *main* female partners	Never	25	25.0	22.5	[2.6–42.4]
	Almost never	27	27.0	27.3	[6.5–48.2]
	Sometimes	15	15.0	3.4	[0.0–15.4]
	Almost always	12	12.0	7.7	[0.0–18.0]
	Always	21	21.0	39.1	[8.3–69.8]
HIV testing (ever; *n=*336)	Never	134	39.9	44.3	[37.0–51.6]
	Once	123	36.6	32.8	[26.4–39.2]
	More than once	79	23.5	22.9	[17.3–28.6]
Considered vaginal sex most “risky” type of sex	Yes	180	53.4	52.8	[45.7–59.9]
Considered anal sex most “risky” type of sex	Yes	59	17.5	15.4	[10.0–20.8]
Considered both equally “risky” types of sex	Yes	119	35.2	35.6	[28.6–42.6]
Ever received information about HIV prevention for sex with men	Yes	75	22.5	18.8	[12.9–24.7]
Ever received information about HIV prevention for sex with women	Yes	183	54.3	53.7	[46.3–61.0]
Knowledge of risk related to positioning	Insertive (top)	56	16.7	15.2	[10.2–20.3]
	Receptive (bottom)	112	33.4	38.0	[30.7–45.3]
	Both carry equal risk	167	49.9	46.7	[39.8–53.6]
Considers safest lubricants to use during anal sex	Petroleum jelly or Vaseline	133	43.6	49.7	[41.2–58.5]
	Water-based lubricant	43	14.1	14.5	[8.0–21.1]
	Others or none	130	42.5	35.6	[27.2–43.9]
Lubricant use	Petroleum jelly or Vaseline	149	45.3	48.3	[40.1–56.0]
	Water-based lubricant	74	22.5	25.2	[18.1–32.3]
	Others or none	106	32.2	26.5	[19.8–33.2]

Several sociodemographic variables were associated with HIV infection in the bivariate analysis (Table 4). These included current age >25 years (RDS-weighted OR: 8.1, 95% CI: 2.9–22.2), single marital status (RDS-weighted OR: 0.2, 95% CI: 0.1–0.4) and having more than one child (RDS-weighted OR: 5.3, 95% CI: 1.8–15.6). Age <16 years at first sex with a man was associated with HIV infection (RDS-weighted OR: 1.7, 95% CI: 0.4–7.5). Considering water-based lubricants to be the safest lubricant (RDS-weighted OR: 0.9, 95% CI: 0.2–3.6) and use of water-based lubricant (RDS-weighted OR: 0.6, 95% CI: 0.2–2.0) were also marginally protective.

**Table 4:** Bivariate and multivariate associations of HIV infection among RDS-recruited MSM in Blantyre, Malawi (August 2011–March 2012; *n=*338)

		HIV negative		HIV positive		Total			OR [unweighted]		OR [RDS weighted]		Multivariate AOR [unweighted]		Mulitvariate RDS-weighted AOR	
									
Variable	Categories	No.	(%)	No.	(%)	No.	(%)	*p*	Estimate	(95% CI)	Estimate	(95% CI)	Estimate	(95% CI)	Estimate	(95% CI)
**Socio-demographics**																
Age	18–25	181	94.2	11	5.7	192	56.8	<0.001	1		1		1		1	
	≥26	105	71.9	41	28.1	146	43.2		6.4	[3.2–13.0]	8.1	[2.9–22.2]	3.2**	[1.3–7.9]	3.9**	[1.2–12.7]
Education	Less than secondary	126	86.3	20	13.7	146	43.2	0.454	1		1		1		1	
	Secondary or higher	160	83.3	32	16.7	192	56.8		1.3	[0.6–2.3]	0.8	[0.3–2.0]	0.6	[0.3–1.4]	0.5	[0.2–1.6]
Employment status	Unemployed	140	88.6	18	11.4	158	46.8	<0.001	1		1		1		1	
	Employed or self-employed	103	75.7	33	24.3	136	40.2		2.5	[1.3–4.7]	2.1	[0.8–5.8]	2.5*	[1.1–5.4]	2.4	[0.9–6.7]
	Student	43	84.7	1	2.3	44	13.0		0.2	[0.1–0.2]	0.2	[0.0–1.5]	0.3	[0.0–2.8]	0.5	[0.0–6.4]
Marital status (with a woman)	Not single, or widowed	35	64.8	19	35.2	54	16.0	<0.001	1		1		1		1	
	Single or never married	251	88.4	33	11.6	284	84.0		0.2	[0.1–0.5]	0.2	[0.1–0.4]	0.7	[0.2–2.0]	0.3*	[0.1–0.8]
Number of children	None	253	88.8	32	11.2	285	84.6	<0.001	1		1		1		1	
	≥1 child	32	61.5	20	38.5	52	15.4		4.9	[2.5–9.6]	5.3	[1.8–15.6]	2.2	[0.7–6.5]	1.2	[0.4–4.1]
Place where respondent grew up	Urban	233	83.5	46	16.5	279	82.5	0.222	1		1		1		1	
	Rural	53	89.8	6	10.2	59	17.5		0.6	[0.2–1.4]	0.7	[0.2–2.4]	0.3	[0.1–1.0]	0.4	[0.1–1.7]
**Sexuality, partnerships and risk exposures**																
Sexual orientation	Gay or homosexual	178	84.8	32	15.2	210	62.3	0.398	1		1					
	Bisexual	106	84.8	19	15.2	125	37.1		1.0	[0.5–1.9]	1.1	[0.5–2.6]				
	Straight or heterosexual	1	50.0	1	50.0	2	0.6		5.6	[0.4–91.2]	24.8	[1.5–420.1]				
Ever disclosed sexual practice to health provider	No	228	85.4	39	14.6	267	79.2	0.414	1							
	Yes	57	81.4	13	18.6	70	20.8		1.3	[0.7–2.7]	1.1	[0.4–3.2]				
Age at first sex with another man	<16 Years old	42	76.4	13	23.6	55	16.3	<0.001	1.6	[0.7–3.8]	1.7	[0.4–7.5]	2.7	[0.9–7.5]	4.3*	[1.2–5.0]
	16–20 Years old	160	92.0	14	8.0	174	51.6		0.5	[0.2–1.0]	0.3	[0.1–0.9]	0.8	[0.3–2.2]	0.6	[0.2–2.2]
	21–25 Years old	62	83.9	12	16.2	74	22.0		1		1		1		1	
	>25	21	61.8	13	38.2	34	10.0		3.2	[0.1–0.4]	3.4	[1.0–11.4]	2.2	[0.7–6.5]	2.6	[0.8–8.6]
Normally has sex with men … in a private home	No	90	79.0	24	21.0	114	33.7	0.039	1		1					
	Yes	196	87.5	28	12.5	224	66.3		0.5	[0.3–1.0]	0.8	[0.3–1.9]				
… in bars or clubs	No	208	86.8	32	13.3	240	71.0	0.102	1							
	Yes	78	79.6	20	20.4	98	29.0		1.7	[0.9–3.1]	0.9	[0.4–2.03]				
… in a hotel	No	225	86.5	35	13.5	260	76.9	0.074	1							
	Yes	61	78.2	17	21.8	78	23.1		1.8	[0.9–3.4]	0.9	[0.4–2.2]				
Condom use at last sex with main male partner	Yes	103	88.0	14	12.0	117	37.0	0.221	1		1					
	No	165	82.9	34	17.1	199	63.0		1.5	[0.8–3.0]	0.9	[0.3–2.6]				
**Social and human rights contexts**																
Feels there are safe places to go to socialize with other MSM	No	88	79.3	23	20.7	111	33.2	0.067	1		1					
	Yes	194	87.0	29	13.0	223	66.8		0.6	[0.3–1.0]	0.4	[0.2–1.1]				
Ever felt afraid to seek health services	No	229	84.8	41	15.2	270	79.9	0.840	1		1					
	Yes	57	83.8	11	16.2	68	20.1		1.1	[0.5–2.2]	0.7	[0.3–2.2]				
Ever in jail	No	227	87.0	34	13.0	261	77.9	0.018	1		1					
	Yes	56	75.7	18	24.3	74	22.1		2.2	[1.1–4.1]	1.4	[0.6–3.4]				
Raped (*N*=337)	No	266	85.5	45	14.5	311	93.3	0.090	1		1		1		1	
	Yes	19	73.1	7	26.9	26	7.7		2.2	[0.9–5.5]	0.6	[0.2–2.0]	2.9	[0.9–9.5]	15	[0.2–9.6]
**Knowledge of HIV risk, prevention methods and practices by HIV diagnosis**																
HIV testing (ever; *n*=336)	Never	113	84.3	21	15.7	134	39.9	0.938	1		1					
	Once	105	85.4	18	14.6	123	36.6		0.9	[0.5–1.8]	0.8	[0.3–2.2]				
	More than once	66	83.5	13	16.5	79	23.5		1.1	[0.5–2.3]	0.5	[0.2–1.5]				
Last 12 months (of ever tested; *N=*202)	Never	79	83.2	16	16.8	95	45.5	0.854	1		1					
	Once	74	86.0	12	14.0	86	41.1		0.8	[0.4–1.8]	0.5	[0.1–1.8]				
	More than once	24	85.7	4	14.3	28	13.4		0.8	[0.3–2.7]	0.5	[0.1–2.3]				
Considered anal sex most “risky” type of sex	No	235	84.2	44	15.8	279	82.5	0.669	1		1					
	Yes	51	86.4	8	13.6	59	17.5		0.8	[0.4–1.9]	0.4	[0.1–1.1]				
Considers safest lubricants to use during anal sex	Petroleum jelly or Vaseline	119	91.5	14	8.49	133	43.5	0.102	1		1					
	Water-based lubricant	104	83.5	26	16.4	130	42.5		1.7	[0.6–4.4]	0.9	[0.2–3.6]				
	Others or none	36	86.7	7	13.2	15	4.9		2.1	[1.1–4.3]	1.2	[0.4–3.2]				
Lubricant use	Petroleum jelly or Vaseline	132	88.6	17	11.4	149	45.3	0.124	1		1		1		1	
	Water-based lubricant	84	79.3	22	20.7	106	32.2		1.5	[0.7–3.3]	0.6	[0.2–2.0]	1.8	[0.7–4.6]	0.9	[0.3–2.4]
	None or others	62	83.8	12	16.2	74	22.5		2.0	[1.1–4.1]	1.3	[0.5–3.4]	2.8*	[1.2–6.5]	2.7	[0.9–8.4]
How many men have you had anal or oral sex with in the past 12 months?													0.9	[0.7–1.1]	0.8	[0.6–1.1]

Exponentiated coefficients; 95% confidence intervals in brackets

* *p*<0.05

** *p*<0.01

analysis sample=318.

The final multivariate model included age, marital status, number of children, knowledge of risk related to positioning (insertive or receptive anal intercourse), lubricant type used, age of first sex with another man, history of rape, number of male anal or oral sex partners and other known confounders such as employment, education and syphilis diagnosis. Of these, current age >25 years (RDS-weighted AOR 3.9, 95% CI: 1.2–12.7), single marital status (RDS adjusted AOR: 0.3, 95% CI: 0.1–0. 8) and age of first sex with a man <16 years (RDS adjusted AOR: 4.3, 95% CI: 1.2–15.0) were independently associated with HIV infection.

## Discussion

This cross-sectional study, the most comprehensive yet conducted among MSM in Malawi, describes the high prevalence of HIV infection as well as the limited uptake of HIV prevention, testing and care services among MSM in Blantyre, Malawi.

HIV prevalence was high among MSM, and nearly all HIV infections were among men who reported being unaware of their status of HIV infection. Only slightly more than half of the population reported ever having been tested, and only half of those were within the last year, potentially explaining this level of undiagnosed HIV infections. Knowing one's status is increasingly more important for HIV prevention. Novel HIV interventions, including pre-exposure prophylaxis for HIV-uninfected men [28,29] and early treatment for people living with HIV [30], represent a new generation of HIV-status-dependent interventions. Awareness of one's HIV status has also been associated with decreased self-reported prevalence of high-risk sexual practices that are associated with HIV transmission [31]. Recent US Centers for Disease Control guidelines have suggested more frequent testing (every 3 or 6 months) based on individual assessment of sexual risk behaviours [32], representing a strategy which may also be relevant for MSM in Malawi.

Young age of first sexual intercourse with a man (<16 years) was independently associated with HIV infection in this population, with almost four times greater odds of HIV infection compared to the referent group. This association may suggest biologic susceptibility during physical development, high-risk sexual behaviours and lack of access to or low use of condoms at a young age, and/or an association with duration of sexual activity [33,34]. Likewise, the association of prevalent HIV infection with older current age may be due to higher cumulative risk exposures for acquisition of HIV. However, estimating the duration of sexual activity is challenging as sexual behaviours are not static, but vary across the life course and as partnerships change [34]. While study-related factors such as low power and potential misclassification of behaviours may partially explain insignificant findings, broader factors such as high background prevalence of HIV in the MSM population [35], biologic susceptibility of rectal mucosa [36] and network-level characteristics may also be more determinative in driving HIV transmission and acquisition risks among these men [35,37]. Nonetheless, this study described a population reporting high-risk behaviours, suggesting the need to ensure accessibility to HIV prevention interventions across ages [38–40]. These behavioural risks, combined with the high proportion of undiagnosed HIV infection in this study, also suggests there is a high likelihood of someone with a high viral load within a sexual network potentially driving onward transmission [41,42]. Future research among MSM in Malawi to better characterize different risk strata among MSM, including reported sexual practices and sexual network characteristics, is needed to better tailor the content of interventions and enable the identification of infection.

While addressing the unique needs of the individual is fundamental, stigma and discrimination have been reported commonly as structural barriers to the uptake of services [43,44]. Experienced and perceived stigma as well as other physical and sexual violence were common among MSM in this study, consistent with earlier quantitative and qualitative studies in Malawi [15,24]. Stigma has been shown to limit health-seeking behaviours and use of HIV prevention methods, disclosure of sexual practices to health providers, and providers’ liberty to provide services to MSM [14,15,24,45,46]. The need to keep male-male partnerships hidden may lead to more frequent, short-term relationships and increased high-risk behaviours [24]. Such responses to stigma and social pressures may explain the high prevalence of concurrency, the high-risk sexual practices reported in this study, the proportion of men who are married or cohabitating with women and the protective effect of single marital status in this analysis. Addressing these social issues is a necessity for improving access to and uptake of effective HIV prevention interventions [8].

Taken together, these data demonstrate that MSM are an underserved and important population for targeted HIV prevention interventions; MSM may specifically benefit from the CHPI that we subsequently developed based on the quantitative results presented here. Mathematical models have shown that high levels of coverage among MSM (i.e., 60–80%) are required to change the trajectory of the HIV epidemic among MSM, and such findings are likely to be relevant in Malawi [2,47,48]. To address low coverage of prevention options among Blantyre MSM and the limitations of single interventions, comprehensive packages of interventions that include behavioural, biomedical and structural approaches may be the most effective approach to reducing HIV among MSM [17]. Such interventions may be feasible in Malawi and may have the same positive impact on sexual transmission that has been observed in other settings, including countries where same-sex practices are criminalized [30,47,49,50].

The method of intervention delivery is critical to the success of HIV prevention programmes in the context of complex social environments. The success of RDS recruitment suggests that interventions leveraging existing peer networks, which have demonstrated efficacy in other settings [51,52], may serve as a feasible approach to providing and supporting HIV prevention interventions for MSM in Malawi. Addressing stigma in healthcare settings may improve provider-patient relationships, facilitate disclosure and meaningful discussion of risk practices, and foster linkage to HIV testing and care [53]. While the subsequent feasibility assessment of the CHPI programme for MSM in Blantyre will be informative for understanding how a comprehensive package may address individual social and behavioural risks for HIV infection, broader social acceptance of MSM may take time and remains a crucial step towards improving the health status of MSM and thus all Malawians [8].

### Limitations

The cross-sectional nature of this study limits the investigation of temporal associations and thus the causality of the exposures and HIV-related outcomes. Additional limitations are related to the ability to fully assess correlates of prevalent HIV infection through behavioural surveys, which may have limited the significance of findings in this study. This may also be amplified by the potential response bias related to asking sensitive questions of a highly stigmatized population. We attempted to address these limitations to the fullest extent possible, including using lifetime and recent recall periods, developing survey questions based on formative research and prior research studies among MSM, and taking measures to ensure the confidentiality and privacy of participants and inform them of these privacy control measures. This study provides equipoise for prospective cohorts of MSM to better characterize HIV incidence and, ultimately, appropriately powered HIV prevention and implementation science studies to assess effective strategies in HIV risk reduction.

There are limitations associated with the use of RDS methodology [54]. Specifically, there is debate around appropriate interpretation of the measures of association and optimal strategies to handle variance in studies using RDS. For example, use of water-based lubricants appeared to be independently protective in the model that did not adjust for RDS, but this association is no longer significant with the introduction of the increased variance associated with RDS adjustment in the model. Despite these analytic challenges, RDS represents a relevant sampling strategy to obtain a diverse sample of a hidden population in the absence of a sampling frame or a sufficient number of established venues [19].

## Conclusions

This study presents an assessment of individual, sexual-network and structural factors and their relationship with prevalent HIV infections among MSM in Blantyre, Malawi. The burden of HIV is high among these men, with the vast majority apparently unaware of their HIV status. Approaches rooted in engagement in the continuum of HIV care will be central moving forward in Malawi [55]. Addressing stigma and discrimination should also represent a core programmatic and policy element of the HIV response, to ensure that these efficacious approaches are translated into effective ones and to optimize the health of MSM living with HIV in Malawi while preventing onward HIV transmission.
